# Relationship between social anxiety and sleep quality in depressed adolescents: the mediating role of internet addiction

**DOI:** 10.3389/fpsyt.2024.1416130

**Published:** 2024-10-10

**Authors:** Yifan Ma, Jingya Li, Meng Zhang, Tiantian Zuo, Linghua Kong, Ying Yang

**Affiliations:** ^1^ Department of Child and Adolescent Mental Behavior, Shandong Mental Health Center, Shandong University, Jinan, China; ^2^ Clinical Psychology, The People’s Hospital of Zouping City, Binzhou, China; ^3^ School of Nursing and Rehabilitation, Cheeloo College of Medicine, Shandong University, Jinan, China

**Keywords:** sleep quality, social anxiety, internet addiction, depression, adolescents

## Abstract

**Objective:**

This study investigated the association between social anxiety and sleep quality and further explored the potential mediating role of internet addiction in depressed adolescents.

**Methods:**

This study included 440 Chinese depressed adolescents (mean age = 15.0 years, SD = 2.0). Participants completed questionnaires, including Social Avoidance and Social Distress Scale, Internet Addiction Test, and Pittsburgh Sleep Quality Index. A structural equation model was used to analyze the association between social anxiety and sleep quality, with internet addiction as a mediator. Gender differences were examined by multi-group analysis.

**Results:**

Among participants, 61.8% reported poor sleep quality. The sleep quality was significantly different on the level of gender (*p* = 0.016), education level (*p* = 0.043), and family history (*p* = 0.002). Sleep quality was positively associated with social anxiety (*p* < 0.001) and internet addiction (*p* < 0.001). Furthermore, internet addiction partially mediated the relationship between social anxiety and sleep quality, and the mediation effect ratio was 16.1% (*p* < 0.001). Finally, no significant differences were found in this mechanism.

**Conclusion:**

We concluded that both social anxiety and internet addiction were risk factors for depressed adolescents’ poor sleep quality. Social anxiety further affected sleep quality through internet addiction.

## 1 Introduction

Poor sleep quality is a global public health problem that has been further exacerbated by the 2019 novel coronavirus disease (COVID-19) pandemic, particularly among adolescents (1, 2). A meta-analysis of 250 studies showed that children and adolescents were the second most affected by poor sleep quality during COVID-19 pandemic, with an estimated prevalence of 45.96% and an increase of at least 20% compared to the pre-pandemic period (3). Compared to healthy adolescents, depressed adolescents are more likely to experience decreased sleep quality, which increases the risks of obesity, diabetes, injuries, poor mental health, attention and behavioral problems, and poor academic performance (4–10). Therefore, it is crucial to identify risk factors that could serve as potential targets for clinical interventions aimed at improving sleep quality in depressed adolescents.

Interpersonal difficulties were common in depressed adolescents, especially social anxiety (11). Social anxiety was a significant risk factor contributing to poor sleep quality, an association that has been described in previous studies (12, 13). Based on cognitive models of Social Anxiety Disorder (SAD), social anxiety leads to post‐event rumination, which subsequently results in a heightened arousal state regarding interpersonal issues (14–16). This process delays individuals’ sleep onset, and consequently negatively affects sleep quality (17). Additionally, during the COVID-19 pandemic, school closures and social isolation reduced opportunities for peer interaction and increased social anxiety (18). This heightened social anxiety likely worsened sleep quality among adolescents. Therefore, we speculated that social anxiety may be associated with sleep quality in depressed adolescents.

Previous studies have shown that internet addiction (IA) was also a potential risk factor for poor sleep quality (19–21). IA was generally referred to a condition in which a person’s internet use was uncontrollable or poorly controlled, accompanied by a high level of attention to or craving for internet content, and leading to serious negative impacts on life or psychological distress, such as poor sleep quality (22, 23). Due to adolescents’ reliance on the internet for education, social interaction, and entertainment during the lockdowns, their risk of developing internet addiction may have significantly increased. The prevalence of IA among adolescents with psychiatric disorders was 31.2% during the COVID-19 pandemic (24). Compared to healthy adolescents, depressed adolescents were found to have a higher level of IA for regulating emotions and obtaining a sense of control. High levels of IA were more likely to induce poor sleep quality (25–27). However, there was limited evidence provided support for the association between IA and sleep quality in the clinical group.

Both social anxiety and IA were risk factors for sleep quality. There was also a postulated link between these two risk factors. According to the Social Compensation hypothesis, the lower level of social pressure on the internet might encourage the self-expression of adolescents with social anxiety, and help them develop online friendships (28). In other words, high levels of social anxiety may induce more IA problems. A study of 370 university students found that social anxiety was associated with problematic social media use (a specific form of IA; Ahmed et al., (29)). For depressed adolescents, a one-year follow-up study found that they were also more likely to prefer friendships on social networks and to use the internet to regulate their emotions and update their negative status (30). Thus, depressed adolescents with higher levels of social anxiety were prone to use the internet to meet their interpersonal needs and therefore had a higher risk of IA. As mentioned earlier, both social anxiety and internet addiction could affect sleep quality; thus we speculate that social anxiety may lead to poor sleep quality through the pathway of IA.

Previous studies have suggested that there may be gender differences in risk factors for depressed adolescents (31, 32). Theories regarding gender differences and sleep quality have suggested that depressed girls suffer more from poor sleep quality (33–36), indicating that girls may be more vulnerable to some certain risk factors. In general, girls spend more time communicating with friends on their mobile phones, which can lead to staying up late (37). This may be a potential approach to dealing with social anxiety. Therefore, girls may be more affected by social anxiety, which could lead to sleep problems mediated by IA.

Poorly previous studies using clinical samples have investigated the relationship between social anxiety, IA, and sleep quality. Thus, this study focused on adolescents with depression and aimed to determine (a) whether social anxiety and IA are related to sleep quality; (b) whether IA plays a mediating role in these associations; and (c) whether there are significant gender differences in this mechanism. This study could provide scientific evidence for developing intervention strategies to improve sleep quality in depressed adolescents.

## 2 Methods

### 2.1 Participants and procedures

This cross-sectional survey was conducted at Shandong Mental Health Center, affiliated with Shandong University, from September 2021 to September 2022. Localized outbreaks and lockdowns of COVID-19 continued to occur during data collection. The inclusion criteria were as follows: (a) The depression diagnosis met the diagnostic criteria for DSM-5 depressive disorders; (b) Their age ranged from 10 to 19 years old. The exclusion diagnostic criteria were as follows: (a) Those are comorbid with other psychiatric disorders, such as neurodevelopmental disorders (autism spectrum disorder, attention deficit hyperactivity disorder, etc.). (b) Those diagnosed with severe physical illness. (c) Those who did not comprehensively complete the relevant questionnaires and scales. 517 participants were asked to complete the questionnaire independently at the hospital and the questionnaires were collected by the investigators on site, 440 of them finally completed all the questionnaires, and the effective return rate was 85%. The final sample included 132 males (30.0%) and 308 females (70.0%), with ages ranging from 10 to 19 years (mean = 15.0; SD = 2.0). This study was approved by the Ethics Committee of Shandong Mental Health Center and informed consent was obtained from all participants.

### 2.2 Measurements

#### 2.2.1 Demographic variables

A self-designed questionnaires was used to collect information on age, gender (boy/girl), education level (primary school and below/secondary school/high school/university and above), family history of depression (yes/no), residence (urban/rural), single-child family (yes/no), marital status of parents (married/divorced/widowed), and annual family income level (higher/average/lower) of the enrolled participants.

#### 2.2.2 Social anxiety

The Social Avoidance and Social Distress Scale (SADS) is an instrument that is widely used to measure social anxiety, developed by Watson and Friend (38). It contains 28 items and is divided into 2 subscales: social avoidance (e.g., “I try to avoid situations, which force me to be very sociable”) and social distress (e.g., “I often find social occasions upsetting”. Scores range from 0 to 28 on a “yes” and “no” scale, with higher scores indicating higher levels of social avoidance and distress. The SADS has been demonstrated to have adequate internal consistency, reliability, and validity among Chinese people (39). The Cronbach’s α is 0.94.

#### 2.2.3 Internet addiction

The Internet Addiction Test (IAT) for adolescents has 20 questions (40). Respondents answer items questions on a 5-point scale (1 = “not at all” to “5 = always”) and scores can range from 20-100. IAT has 3 subscales (41), emotional and cognitive internet preoccupation-reliance on online life (e.g., “How often do you try to hide how long you’ve been online”), neglecting work and lack of self-control (e.g., “How often do you find that you stay online longer than you intended”), and social problems (e.g., How often do you prefer the excitement of the internet to intimacy with your partner”). The IAT has been validated in Chinese adolescents (42). In the sample of this study, the Cronbach’s alpha coefficient was 0.93.

#### 2.2.4 Sleep quality

Pittsburgh Sleep Problems Index (PSQI) consists of 18 items (e.g., “During the past month, what time have you usually gone to bed at night”) and 7 subscales: subjective sleep quality, sleep latency, sleep duration, habitual sleep efficiency, sleep disturbances, use of sleeping medications, and daytime dysfunction (43). The total score ranges from 0 to 21, and scores higher than 5 indicate poor sleep quality. Higher scores suggest poorer sleep quality. The PSQI has been demonstrated good reliability among Chinese populations (44), and has been extensively used among Chinese adolescents (45). The Cronbach’s alpha coefficient was 0.79 in the sample of this study.

### 2.3 Data analyses

Analyses were conducted using SPSS (26.0) and Mplus (8.0). Descriptive statistics were performed to describe the demographic characteristics of the study sample. Pearson correlation analyses were run to examine the correlations of sleep quality with social anxiety, and internet addiction.

Structural equation modeling (SEM) was conducted to examine the mediation effect of IA on the association between social anxiety and sleep quality. Latent variables for social anxiety, IA, and sleep quality were calculated using subscales as indicators. Age, education level and family history of depression were covariates. The model fit was evaluated based on χ2/df (≤ 3), the comparative fit index (CFI > 0.90), the Tucker-Lewis index (TLI > 0.90), the standardized root means square residual (SRMR < 0.08), and the root mean square error of approximation (RMSEA < 0.08). The bootstrap analysis was used to examine the mediation effects with 5000 bootstrap samples. A two-sided *p*-value < 0.05 was considered to indicate significant differences. Multi-group analysis was utilized to evaluate gender differences.

Considering that all variables were reported by the participants, we used Harman’s single-factor test to examine the common method variance (46). It showed that 18 factors with eigenvalues greater than one were extracted, with Factor 1 accounting for 25.68% of the variance (less than 40%). These results suggest that common method variance is not of great concern in the present study.

Sensitivity analyses were performed to ensure the robustness of our results. We employed multi-group analysis to explore potential differences between education levels. Furthermore, we established theoretically plausible alternative models representing competing hypotheses (47).

## 3 Results

Descriptive analyses showed differences in PSQI score among the demographic characteristics in **Table 1**. The mean age was 15 years (SD = 2.0). Significant differences with PSQI score were observed in gender (*t* = - 2.42, *p* = 0.016), education level (*F* = 2.74, *p* = 0.043), and family history (*t* = 3.10, *p* = 0.002). The description of PSQI score and subscale scores was shown in **Table 2**. The mean PSQI score was 7.88 (SD = ± 4.77), and 61.8% of participants reported poor sleep quality (PSQI score > 5).

**Table 1:** Demographic characteristics of sample and differences in PSQI score (N= 440).

Variables	n(%)/(M ± SD)	PSQI score (M ± SD)	t/F	P
Age	15.0 ± 2.0	7.8 ± 4.77		
Gender			-2.42	0.016
boy	132(30.0)	7.05 ± 4.89		
girl	308(70.0)	8.24 ± 4.69		
Education level			2.74	0.043
primary school and below	22(5.0)	6.09 ± 4.52		
secondary school	195(44.3)	8.04 ± 5.03		
high school	190(43.2)	8.22 ± 4.62		
university and above	33(7.5)	6.24 ± 3.77		
Family history of depression			3.10	0.002
yes	52(11.8)	9.79 ± 5.32		
no	388(88.2)	7.63 ± 4.65		
Resident			0.44	0.660
urban	294(66.8)	7.95 ± 4.70		
rural	146(33.2)	7.74 ± 4.94		
Single-child family			-1.29	0.196
yes	164(37.3)	7.50 ± 4.53		
no	276(62.7)	8.11 ± 4.91		
Marital status of parents			1.08	0.342
married	386(87.7)	7.89 ± 4.78		
divorced	39(8.9)	8.38 ± 4.97		
widowed	15(3.4)	6.27 ± 4.08		
Income (¥)			1.52	0.220
higher (>150,000)	94(21.3)	7.13 ± 4.44		
average(50,000-150,000)	244(55.5)	8.05 ± 4.76		
lower (<50,000)	102(23.2)	8.18 ± 5.08		

PSQI, Pittsburgh sleep problems index.

M ± SD, mean ± standard deviation.

**Table 2:** Seven subscales of sleep quality in depressed adolescents (N = 440).

Component of sleep quality		Frequency	%
Subjective sleep quality (subscale 1)	very good	94	21.4
	good	192	43.6
	Bad	102	23.2
	very bad	52	11.8
Sleep latency (subscale 2)	0 (none)	108	24.5
	1-2 (low)	128	29.1
	3-4 (middle)	116	26.4
	5-6 (high)	88	20.0
Sleep duration (subscale 3)	>7 hours	244	55.5
	6–7 hours	70	15.9
	5–6 hours	98	22.3
	<5 hours	28	6.4
Habitual sleep efficiency (subscale 4)	>85%	283	64.3
	76–85%	78	17.7
	66–75%	32	7.3
	<65%	47	10.7
Sleep disturbances (subscale 5)	0 (none)	39	8.9
	1–9 (low)	214	48.6
	10–18 (middle)	143	32.5
	19–27 (high)	44	10.0
Use of sleeping medication (subscale 6)	0 (none)	271	61.6
	1 (1 time/week)	33	7.5
	2 (2-3 times/week)	23	5.2
	3 (≥3 times/week)	113	25.7
Daytime dysfunction (subscale 7)	0 (none)	96	21.8
	1–2 (low)	149	33.9
	3–4 (middle)	125	28.4
	5-6 (high)	70	15.9
PSQI score (7.88 ± 4.77)	0–5	168	38.2
	>5	272	61.8

PSQI, Pittsburgh sleep problems index.

The bivariate correlations were presented in **Table 3**. All PSQI subscales were positively related to all SADS subscales (r: 0.15 to 0.48, all p < 0.01). All SADS subscales were statistically significantly correlated with all IAT subscales (r: 0.28 to 0.41, all p < 0.001). The PSQI subscales were found to be significantly correlated with IAT subscales (r: 0.10 to 0.34, all p < 0.05), except for the correlation between C4 (habitual sleep efficiency) and all IAT subscales and the correlation between C6 (use of sleeping medication) and I2 (neglecting work and lack of self-control).

**Table 3:** Correlations between sleep quality, social anxiety and internet addiction (N = 440).

Variables	1	2	3	4	5	6	7	8	9	10	11	12
Sleep quality												
1.C1	1											
2.C2	.624^***^	1										
3.C3	.445^***^	.336^***^	1									
4.C4	.310^***^	.313^***^	.480^***^	1								
5.C5	.596^***^	.570^***^	.281^***^	.227^***^	1							
6.C6	.355^***^	.369^***^	0.09	.160^**^	.340^***^	1						
7.C7	.549^***^	.494^***^	.329^***^	.166^***^	.550^***^	.273^***^	1					
Social anxiety												
8.S1	.409^***^	.335^***^	.225^***^	.133^**^	.432^***^	.185^***^	.480^***^	1				
9.S2	.388^***^	.337^***^	.223^***^	.151^**^	.445^***^	.196^***^	.477^***^	.845^***^	1			
Internet addiction												
10.I1	.325^***^	.251^***^	.155^**^	.041	.309^***^	.099^*^	.336^***^	.355^***^	.386^***^	1		
11.I2	.301^***^	.216^***^	.171^***^	.029	.238^***^	.033	.337^***^	.280^***^	.317^***^	.800^***^	1	
12.I3	.326^***^	.251^***^	.206^***^	.052	.312^***^	.162^**^	.342^***^	.369^***^	.409^***^	.736^***^	.669^***^	1

^*^ *p*< 0.05; ^**^ *p* < 0.01; ^***^ *p* < 0.001.

C1, subjective sleep quality; C2, sleep latency; C3, sleep duration; C4, habitual sleep efficiency; C5, sleep disturbances; C6, use of sleeping medication; C7, daytime dysfunction. S1, social avoidance; S2, social distress. I1, emotional and cognitive internet preoccupation-reliance on online life; I2, neglecting work and lack of self-control; I3, social problems.

**Figure 1:**
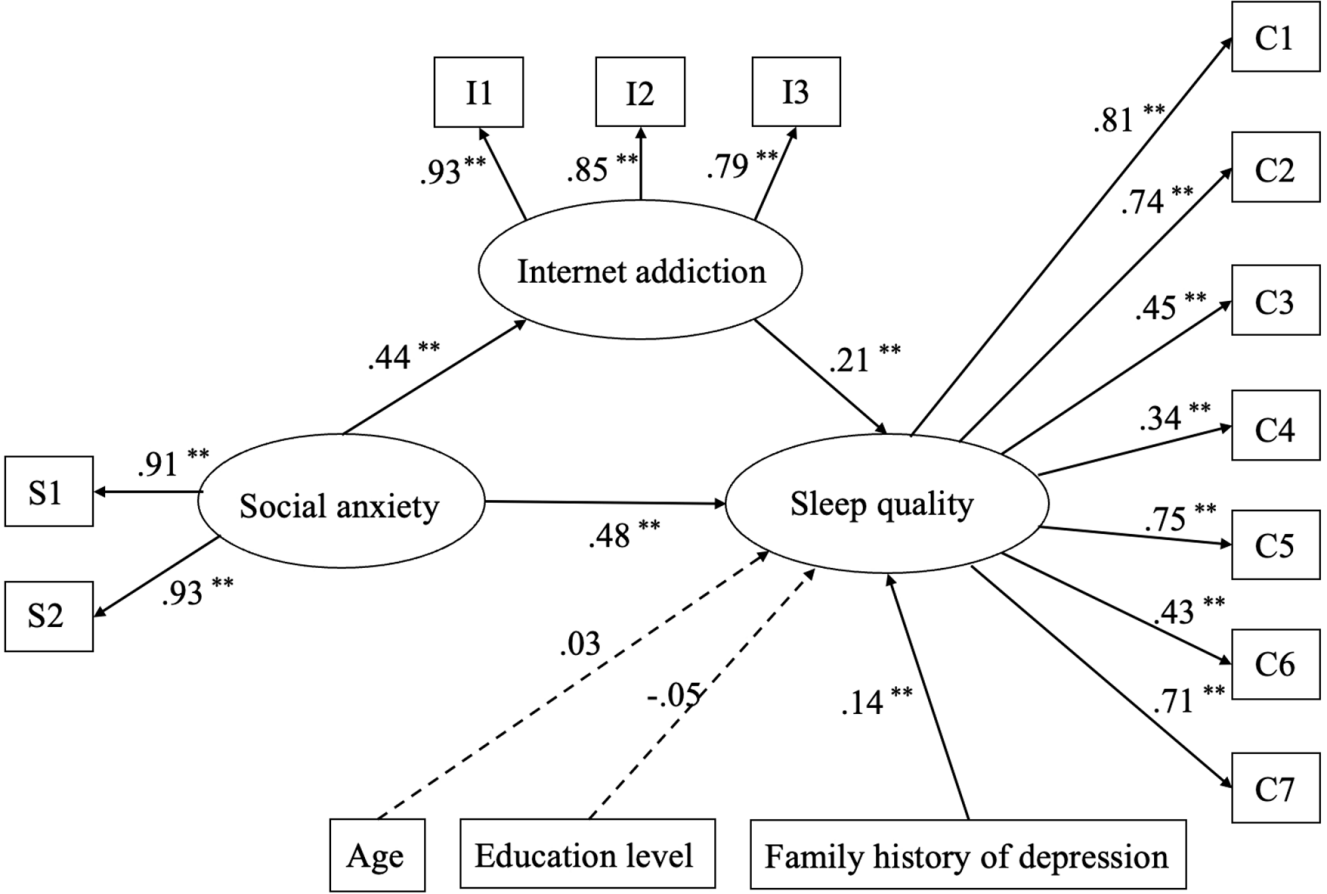
The mediation model of internet addiction on social anxiety and sleep quality (N = 440). The fit indices for the modified model were acceptable: χ2 = 256.544, df = 99, χ2/df = 2.591, CFI = 0.940, TLI = 0.929, RMSEA = 0.060, and SRMR = 0.055. C1, subjective sleep quality; C2, sleep latency; C3, sleep duration; C4, habitual sleep efficiency; C5, sleep disturbances; C6, use of sleeping medication; C7, daytime dysfunction. S1, social avoidance; S2, social distress. I1, emotional and cognitive internet preoccupation-reliance on online life; I2, neglecting work and lack of self-control; I3, social problems. **P<0.01.

The mediation model of IA and the standardized coefficients for each variable are shown in **Figure 1**. The SEM depicted significant regression and correlation paths, with all the path coefficients statistically significant at the level of p < 0.01, except for gender and education level. The fit indices for the model were acceptable: χ2 = 256.544, df = 99, χ2/df = 2.591, CFI = 0.940, TLI = 0.929, RMSEA = 0.060, and SRMR = 0.055.

According to this model, social anxiety and IA were significant predictors of sleep quality. The standardized direct effect value for social anxiety on sleep quality was 0.48 (*p* < 0.001, 95% CI [0.39, 0.57]) and the standardized direct effect value for IA on sleep quality was 0.21 (*p* < 0.001, 95% CI [0.11, 0.31]). Social anxiety had a significant predictive effect on IA, and the standardized direct effect value was 0.44 (*p* < 0.001, 95% CI [0.36, 0.51]). The standardized indirect effect value between social anxiety and sleep quality via IA was 0.092. The standardized total effect value of social anxiety on sleep quality was 0.572. The mediated effect ratio was 16.1%. However, no gender differences(Δχ2/df = 1.04, *p* < 0.05) were found in this SEM. Constraining each of the paths to be equal across gender appeared to be reasonable. These results suggest that the IA factors function similarly across the two genders.

The sensitivity analysis results showed no differences between education levels in the model (**Supplementary Figures S1**, **S2**). Alternative models showed acceptable fits (**Supplementary Table S1**, **Supplementary Figures S3**, **S4**); however, their fit indices did not surpass those of our model, and our model retained lower AIC and BIC values (48). These results strengthen the reliability of our findings.

## 4 Discussion

The cross-sectional study, involving 440 depressed Chinese adolescents found that social anxiety and internet addiction were associated with sleep quality after adjustment for gender, education level, and family history of depression. In addition, this study indicated that internet addiction partly mediated the relationship between social anxiety and sleep quality. No gender differences were found in this mechanism.

In this study, 61.8% of participants reported poor sleep quality, which was consistent with the prevalence in clinical samples and higher than in community samples (17, 49, 50). Adolescents with depressive symptoms who usually have low levels of emotional regulation ability tend to prolonged sleep latency and poor sleep quality (51, 52). In addition, our study found girls, studying in high school, and with a family history of depression were more likely to develop poor sleep quality. According to the Diathesis-stress theory (53), the family history of depression is a diathesis factor, which may render individuals to be more susceptible to environmental influences that induce poorer sleep quality. Furthermore, academic stress as a stressor would chronically increase the hypothalamus–pituitary–adrenal (HPA) axis activity and higher cortisol production, which deteriorates sleep quality (54, 55). In the Chinese traditional view, academic performance is paramount for entering university, so high school students face significant academic stress from parents, teachers, and peers, which induces poor sleep quality (56). Moreover, girls in puberty experience more hormonal disruption and endocrine dyscrasia affecting their sleep quality (57).

Our results indicated that social anxiety was directly related to sleep quality in depressed adolescents. The pathway may be explained by the process of rumination, which involves negative over-thinking and could amplify and prolong negative emotional states (58, 59). Depressed adolescents with social anxiety were more likely to engage in rumination, usually manifested as overthinking before sleep, which exacerbated their anxious emotions. Therefore, they may suffer from insomnia and have reduced sleep quality. Additionally, serotonin and dopamine, both neurotransmitters, were key factors in the generation and development of depression and were also associated with social anxiety and sleep quality (60, 61). It has been claimed that social anxiety, along with unbalanced concentrations of serotonin and dopamine in the brain would lead to poor sleep quality by affecting the sleep-wake cycle (62, 63). Adolescents with depression had high levels of social stress and social anxiety, which can result in physiological changes, such as increased activity in the stress system and the autonomous nervous system, and eventually disrupted sleep patterns and lead to poor sleep quality (64–66).

Social anxiety can also indirectly affect sleep quality through IA. This result supported the cognitive-behavioral model of internet addiction (67). Adolescents with social anxiety usually generate maladaptive cognitions (e.g., “I am a failure when I am offline”, “the internet is my only friend”) that can further induce uncontrolled internet use, even IA. Furthermore, IA was related to evening circadian preference (ECP), a behavioral predilection for later sleep and wake timing, associated with later biological circadian timing (68). Depressed adolescents with IA who usually surf the internet at night, and would shorten their sleep duration, leading to misalignment with their biological circadian timing, and would be prone to disturbed or insufficient sleep and reduced sleep quality. Misalignment between biological circadian timing and sleep-wake behavior is a potential mechanism of depression (69). It may further exacerbate “failure” cognitions of social anxiety in depressed adolescents. This would create a vicious circle from social anxiety to depression that can be a major obstacle to treatment, illustrating the importance of breaking this vicious circle for both treatment and intervention to improve sleep quality in depressed adolescents.

Finally, no gender differences were found in this mechanism, which was inconsistent with our assumptions. A possible explanation was that many risk factors can influence sleep quality, and there may be other pathways affecting gender differences in sleep quality in depressed adolescents, such as sex hormones and stress (70). Future research is needed to explore these pathways further.

There were several limitations in this study. First, this was a cross-sectional study. We only explored potential mechanisms of the relationship between social anxiety, internet addiction, and sleep quality based on theoretical foundations. The directionality and causality of the relationships were not included. Longitudinal studies should be carried out to address this issue. Second, the data were based on subjective reports, potentially entailing recall or social desirability bias. Future research could use objective measurement tools (e.g., actigraphy, polysomnography) in order to enhance the reliability and validity of the research. Third, these findings were limited to depressed adolescents. Further investigation is still required to test these findings among other groups. Fourth, this study did not control for the effects of medication use among the participants, which could introduce bias and impact the accuracy of our results. Future research should account for medication effects to better understand the relationships between social anxiety, sleep quality, and internet addiction. Fifth, this study only focuses on internet addiction as a mediator, neglecting other potential factors such as rumination, family dynamics, social support, or coping mechanisms. Future research should include these factors for a more comprehensive understanding. Lastly, reliable biological indicators such as serotonin and dopamine can be measured in future studies to explore the mechanism further. Despite these weaknesses, this study had several strengths. We focused on the neglected group of depressed adolescents who were suffering from poor sleep quality. In addition, this study investigated the potential mechanism in the relationship between social anxiety and sleep quality. Moreover, it provided a theoretical foundation for clinical prevention and interventions to enhance sleep quality. Psychiatrists, psychologists, and public health institutes can target social anxiety and internet addiction and take intervention measures to improve sleep quality among depressed adolescents.

## 5 Conclusions

In summary, our results provided evidence of significant associations between social anxiety, internet addiction, and sleep quality in depressed adolescents. We found significant indirect effects of social anxiety on sleep quality through internet addiction. These findings have both theoretical and practical implications for the intervention and treatment of poor sleep quality in depressed adolescents.

## Data Availability

The raw data supporting the conclusions of this article will be made available by the authors, without undue reservation.
