# Supplementary material for: Relationship between social anxiety and sleep quality in depressed adolescents: the mediating role of internet addiction
Source: Front Psychiatry. 2024 Oct 10;15:1416130. doi: 10.3389/fpsyt.2024.1416130 (PMC11503650; doi:10.3389/fpsyt.2024.1416130)
Supplement: Supplementary file 1 [file DataSheet1.docx]

***Supplementary Material***

**Relationship between social anxiety and sleep quality in depressed adolescents: the mediating role of internet addiction**

1. Multi-group analysis was utilized to evaluate differences in education levels. The fit indices for the model were acceptable: χ2 = 297.199, df = 167, χ2 / df =1.780, CFI = 0.950, TLI = 0.947, RMSEA = 0.060 and SRMR = 0.061.


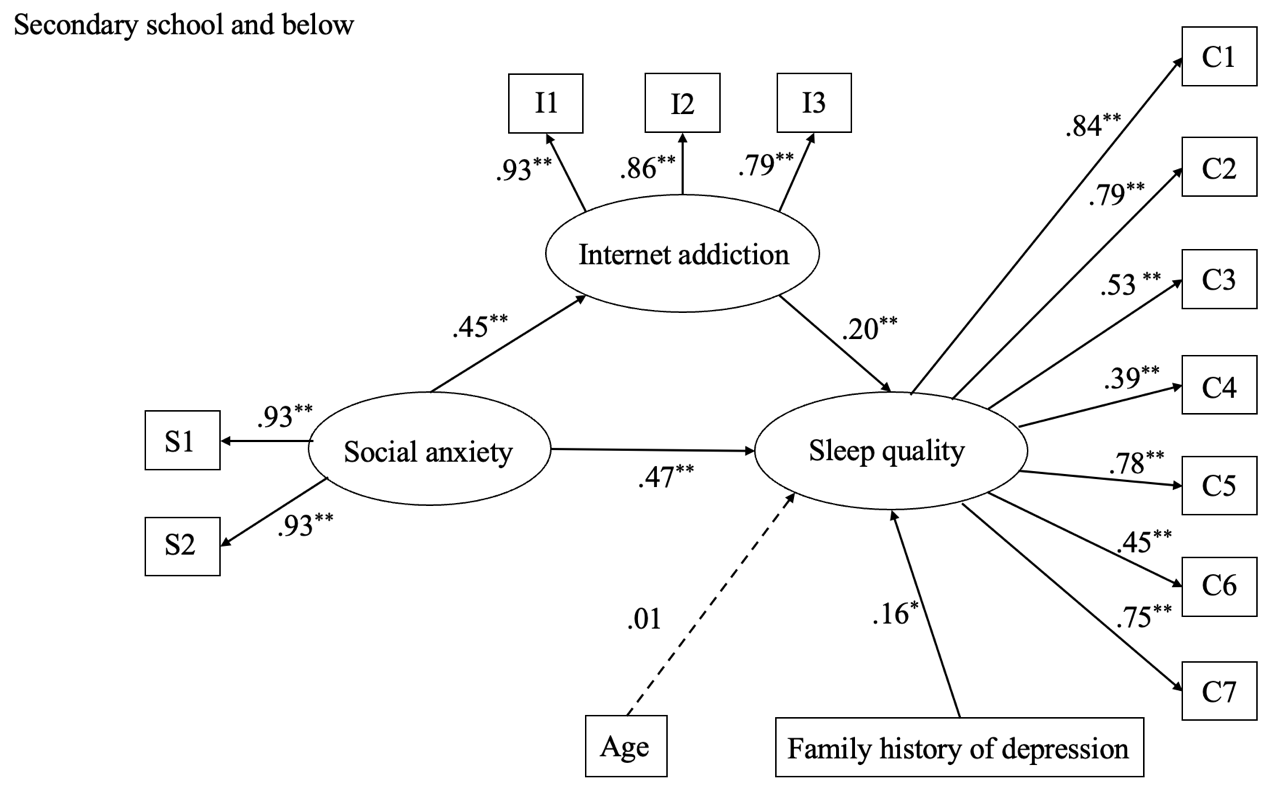


**Figure S1.** The mediating effects model in the Secondary school and below group. ^⁎^p < .05, ^⁎⁎^p < .01.

C1, subjective sleep quality; C2, sleep latency; C3, sleep duration; C4, habitual sleep efficiency; C5, sleep disturbances; C6, use of sleeping medication; C7, daytime dysfunction. S1, social avoidance; S2, social distress. I1, emotional and cognitive internet preoccupation-reliance on online life; I2, neglecting work and lack of self-control; I3, social problems.


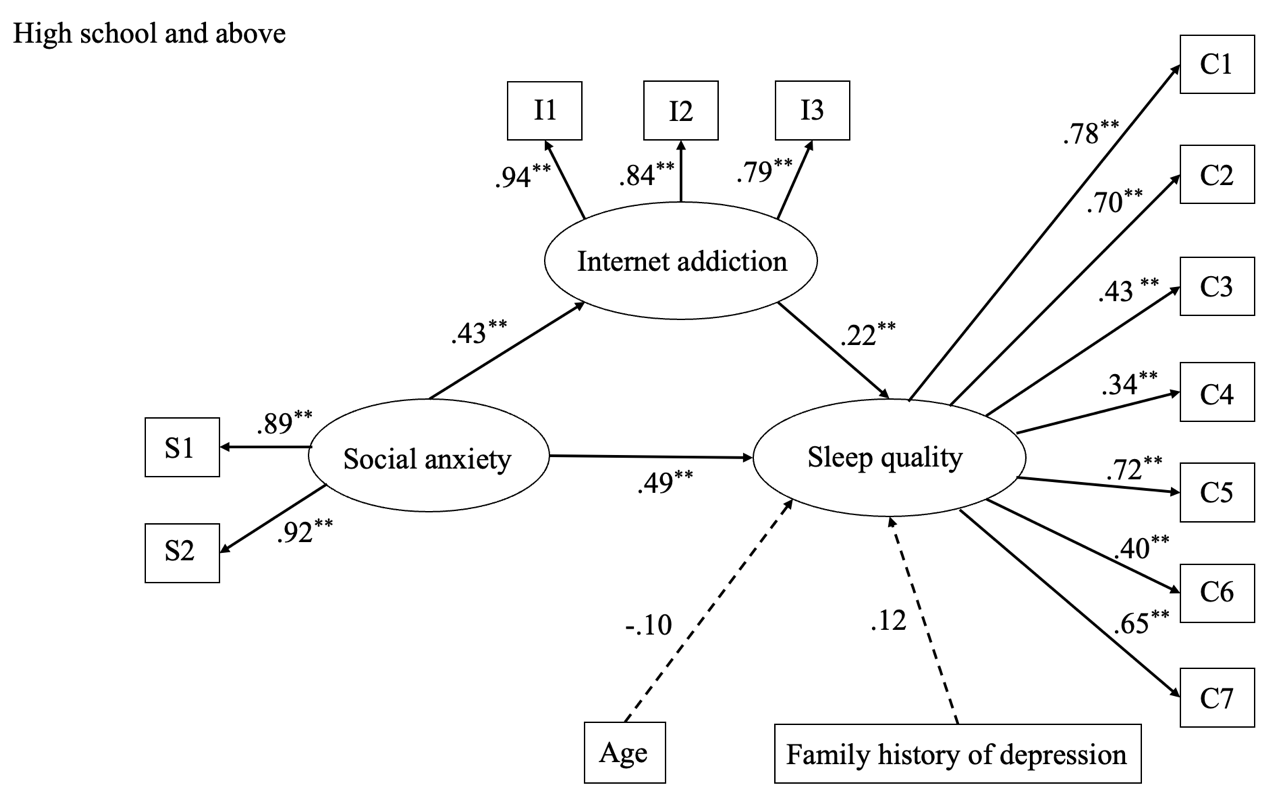


**Figure S2.** The mediating effects model in the High school and above group. ^⁎^p < .05, ^⁎⁎^p < .01.

C1, subjective sleep quality; C2, sleep latency; C3, sleep duration; C4, habitual sleep efficiency; C5, sleep disturbances; C6, use of sleeping medication; C7, daytime dysfunction. S1, social avoidance; S2, social distress. I1, emotional and cognitive internet preoccupation-reliance on online life; I2, neglecting work and lack of self-control; I3, social problems.

2. Model fits and Structures of alternative models.

**Table S1.** Model fits of the models.

| Model | χ2 | df | χ2 / df | CFI | TLI | RMSEA | SRMR | AIC | BIC |
| --- | --- | --- | --- | --- | --- | --- | --- | --- | --- |
| Model 1 | 256.544 | 99 | 2.591 | 0.940 | 0.929 | 0.060 | 0.055 | 20474.595 | 20654.413 |
| Model 2 | 256.544 | 99 | 2.591 | 0.940 | 0.929 | 0.060 | 0.055 | 20474.595 | 20654.413 |
| Model 3 | 268.077 | 99 | 2.708 | 0.936 | 0.924 | 0.062 | 0.059 | 20486.128 | 20665.946 |

Model 1: the current model; Model 2: the mediation model of social anxiety on internet addiction and sleep quality; Model 3: the mediation model of social anxiety on sleep quality and internet addiction.
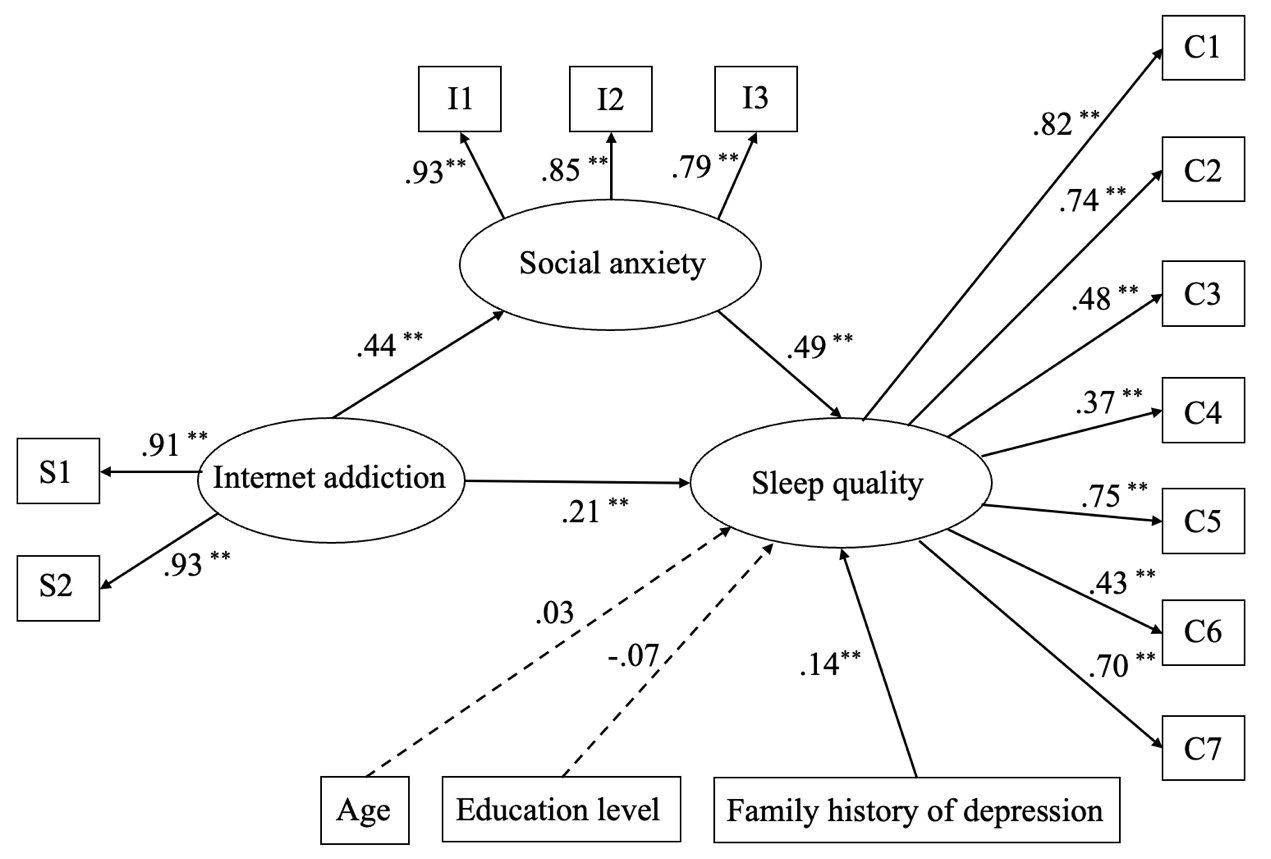
**Figure S3.** The mediation model of social anxiety on internet addiction and sleep quality. ^⁎^p < .05, ^⁎⁎^p < .01. C1, subjective sleep quality; C2, sleep latency; C3, sleep duration; C4, habitual sleep efficiency; C5, sleep disturbances; C6, use of sleeping medication; C7, daytime dysfunction. S1, social avoidance; S2, social distress. I1, emotional and cognitive internet preoccupation-reliance on online life; I2, neglecting work and lack of self-control; I3, social problems.


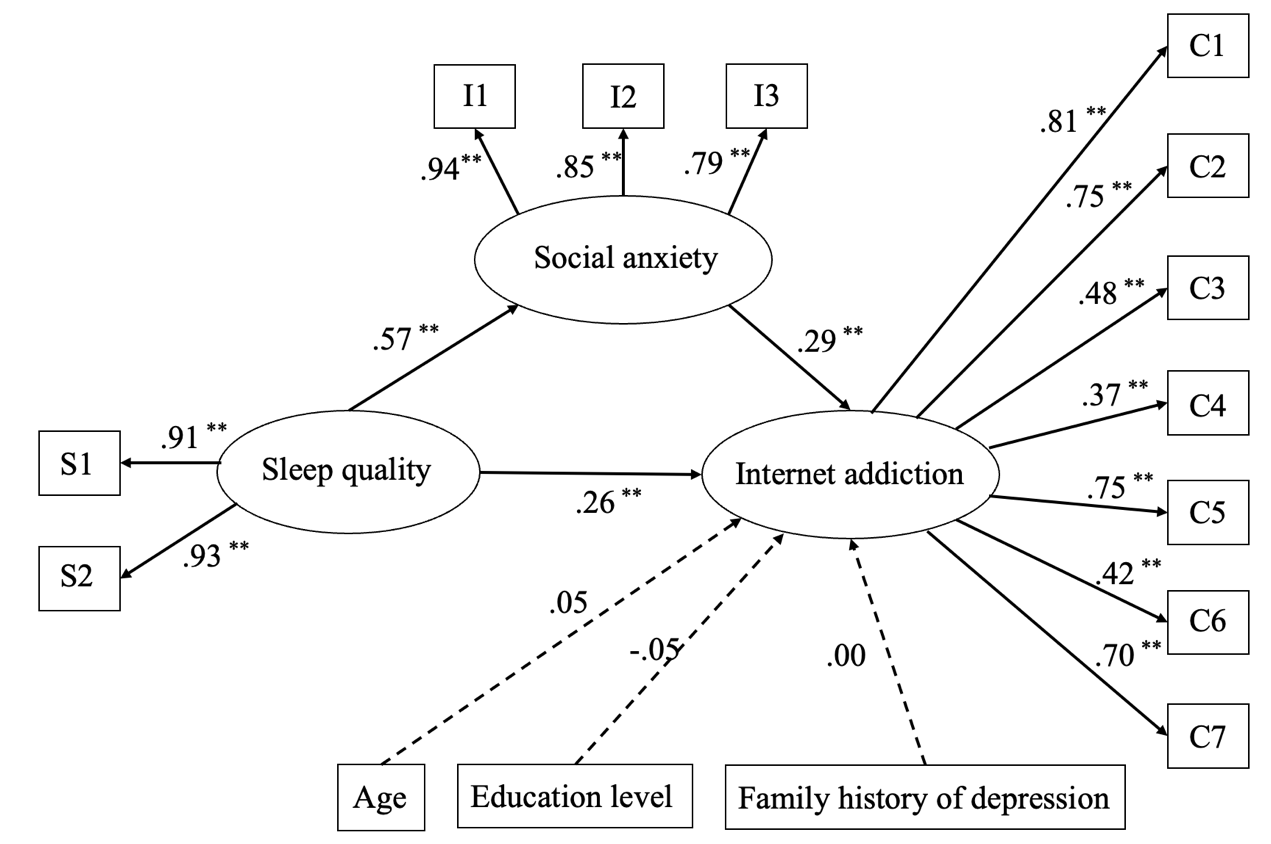
 **Figure S4.** The mediation model of social anxiety on sleep quality and internet addiction. ^⁎^p < .05, ^⁎⁎^p < .01. C1, subjective sleep quality; C2, sleep latency; C3, sleep duration; C4, habitual sleep efficiency; C5, sleep disturbances; C6, use of sleeping medication; C7, daytime dysfunction. S1, social avoidance; S2, social distress. I1, emotional and cognitive internet preoccupation-reliance on online life; I2, neglecting work and lack of self-control; I3, social problems.
